# Supplementary material for: Learning from failure: Simulating pandemic agreement negotiations in a global health classroom
Source: PLOS Glob Public Health. 2025 Aug 27;5(8):e0003661. doi: 10.1371/journal.pgph.0003661 (PMC12385383; doi:10.1371/journal.pgph.0003661)
Supplement: Attachment — Submitted filename: PGH_Response2Reviews.docx [file pgph.0003661.s002.docx]

Dear Editors

Thank you for sharing the reviews of our manuscript. We are pleased to see that both reviewers recognize the merit of the manuscript topic and its value to global health scholars and instructors. We are also grateful for the constructive feedback from both reviewers, and have endeavored to address each point to the degree we are able.

As you requested, we have included an author summary. However, in terms of data availability, the protocol approved by our ethics board does not allow for data to be made publicly available as doing so would compromise participant privacy. It is impossible to deidentify this small qualitative data set to the degree required to make it publicly available. If you have any further questions about this please let me know.

We have responded to each of the reviewers’ comments below. Thank you again for considering our article for publication in Plos Global Health.

Sincerely,

Julia

**Reviewer #1**

1. Lack of comprehensive review of related experiences/ “there is no systematic search strategy or in-depth review”

- We think the idea of conducting a systematic review of the literature on serious games as a pedagogical approach is an excellent one, but that is not the aim of this paper, which instead aims to report on the application of specific serious game in a global health class. With this in mind, we have conducted further searches for literature on serious games and global health, and found eight further articles, which we have added, including both a scoping review and meta analysis on the use of serious games in health education. Considering the limited literature specific to global health (here we only found two further articles), which other cited articles also note, we believe the section outlining existing knowledge on serious games as a pedagogical tool covers relevant themes in the literature to the degree necessary for contextualizing our study, as is common in research papers.

2. Methodological concerns as a qualitative study:

“Methodology: The study lacks a defined qualitative methodology, which is a crucial aspect that the authors need to address. With a clear methodological framework, the study's basis and approach to data analysis are adequately justified.”

- We have explicitly noted that our methodology is an exploratory, mixed method qualitative study including participant observation and thematic document analysis

“Coding and analysis process: The thematic analysis needs a more detailed explanation of the coding process, including steps for ensuring reliability, which are essential for robust qualitative findings.”

- We have added detail on the thematic analysis, including referencing similar methodological approaches.

“Role of observers and bias management: Given the authors' observational role within the simulation, potential biases should be addressed. The manuscript would benefit from describing measures to maintain objectivity in data collection and analysis.”

- We have explained how we aimed to mitigate bias through co-author discussions, comparing and contrasting observations. We have also highlighted the risk of bias in the limitations section.

3. Small sample size:
“The study is based on a very limited sample, and whether data saturation has been achieved remains to be determined. Although the study offers insights into learning outcomes from the simulation, a larger sample would be necessary to draw reliable and generalisable conclusions.”

- We realise the small sample is a limitation, which we have noted. Unfortunately, there is not much we can do about this due to the size of the class. We hope to be able to conduct more robust studies of serious games with larger cohorts in future. We further note a number of other studies using a similar sample size and method (see for example Covvey et al 2017 and Talantikite et al. 2020)

4. Absence of a comparison group:
“A comparison between the serious game approach and more traditional teaching methods (e.g., lecture-based or practical sessions without gamification) would strengthen the study's claims about the unique effectiveness of serious games in this context. Such a comparison would help clarify the added value of gamification versus conventional approaches in achieving learning objectives.”

- As this is an exploratory qualitative study, which we have now clarified in the methods, it is not intended to measure specific learning outcomes. Therefore, we do not think comparison would be an appropriate methods, and of course this is not possible to do retroactively. We further note that very few published assessments of serious game referenced here use a comparison group, likely for the reasons we note above.

“In its current form, this manuscript does not yet serve as a rigorously peer-reviewed piece of evidence for the field. With further development, it could make a valuable contribution, yet at this stage, it may be better suited as a "Correspondence" or "Letter to the Editor" rather than a full original research article, given its lack of necessary components for a robust qualitative study.”

- We realise this is somewhat of a unique, exploratory article with a small sample. If the editors think it would be better placed as a commentary or another article type, we are open to their suggestions.

**Reviewer #2**

“Pg2 line 34. Add ‘were’ before ‘developed out of the tradition..’. There are few other typos/ missing words so a thorough read should do take care of this error.”

- we have added ‘were’ and proofread for typos

“Pg2 lines 46-54 are part of methodology and can be moved there.”

- this paragraph (starting this articles aims to . . .) states the purpose of the paper and so we feel it must remain in the introduction

“Pg 3 lines 63-80. Overall, a significant chunk of this information and other country examples can be moved to discussion section.”

- Our purpose with this section was to briefly outline existing evidence on use of serious games. However, we realise this was not clearly presented. We have aimed to rectify this with a subtitle and revisions to the text.

“Pg3. Line 106-107 appears very abruptly after establishing the need of application of serious games/ simulation in global health teaching, without stating the width or depth of existing knowledge or any knowledge gap.”

- We have added a sentence to the end of this section summarizing status of existing knowledge and knowledge gap.

“Pg 6 Line 127. Are there other games where fictional world settings have been used to simulate experiential learning, specially with curriculum of subjects like global health, which is very embedded in the current ‘global situation’ and its unique history and geography. A stronger rationale is required to justify the fictional world setting.”

- we have referenced another game using a fictionalized context and expanded on this point further.

“I feel that the introduction and methodology of the game Accord can be a part of the methods and materials, starting with description of the game, pre- and post-surveys etc. Methods section would not be the place where limitations or pluses of a methodology are discussed. Instead, these should be parked in the limitation section of the article. At the moment all this content, while coherent, is moving to and fro, and would benefit from a little bit of restructuring for better flow and removal of repetitions.”

- Thank you for these suggestions. We have reorganized as suggested

“It would make for a catchier read if the results and students’ reflections from Pg 12 Lines 245 to Page 21 lines 496 are structured as per emerging themes based on the thematic analysis undertaken. Also, even though thematic analysis is mentioned, it would be valuable to elaborate on the emerging themes as well as the reason behind extracting these themes for the study or study objective. At the moment no themes have been discussed, which makes one wonder about the rationale of choosing to do thematic analysis, and the utility of this analysis.”

- We apologise for lack of clarity around our approach to thematic analysis. The learning objectives as well as additional learning points in Table 1 are the themes we analyzed for. We have aimed to clarify this approach as well as the reasons for it in the methods section, as well as at the start of the results’ sub-sections pertaining to the themes.

“Finally, a succinct summary of results, key learning outcomes and takeaways would nicely close the article, which can then be followed by conclusion. The takeaways should also include how the failures in the game have contributed to the learning curve of the students, aligned with or deviated from the desired learning outcomes, and how can similar models be developed and used.”

- We have added this summary to the discussion

“In the end, coming towards the title of the article, the quote ‘I wanted to be a responsible public health professional’ somehow does not seem to effectively convey what the article is about. I would suggest titles indicating simulation, or serious games in action, or using them in wicked global health challenges, to develop critical thinking and cooperation and collaboration skills.”

- Thank you for this suggestion, we have revised the title
