# Supplementary material for: Learning from failure: Simulating pandemic agreement negotiations in a global health classroom
Source: PLOS Glob Public Health. 2025 Aug 27;5(8):e0003661. doi: 10.1371/journal.pgph.0003661 (PMC12385383; doi:10.1371/journal.pgph.0003661)
Supplement: Attachment — Submitted filename: Accord_Response2Reviews2.docx [file pgph.0003661.s003.docx]

Dear Editor and Reviewers

Thank you for the helpful and constructive feedback on our paper. We appreciate the time and thought you have invested and have done our best to respond to each point raised below.

Sincerely,

Julia Smith, Ellie Gooderham and Julianne Piper

**Reviewer 3 suggestions:**

“Introduction: highlighting the wider range of Model World Health Assemblies that have been launched in recent years, bridging Model UN activities and this mock Pandemic Agreement negotiation.”

- Thank you for pointing us to MWHAs that have been held a Chapel Hill. We have noted these and the paper in the introduction as well as made further refeernce to the Model World Health Assemblies (see lines 201-213)

“Methods: discussing further how class discussions drew from the global health diplomacy literature to prepare them for Accord exercise.”

- We have added an explanation that students had to draw on course materials to prepare the articles for the negotiation, as well as for the reflective essay. (see lines 299-311)

“Discussion: considering how -- now that the pandemic agreement is complete -- students could engage with a past policy reforms, comparing their negotiations with the actual diplomacy in the World Health Assembly.”

- We have added mention of completed pandemic agreement etc in the discussion. (see lines 774-783)

**Reviewer 4 suggestions:**

The reviewer notes the improvements made with the previous revision and points out that the article, “is largely a reflection on the pedagogical benefits of the particular exercise . . .as opposed to a discussion of the benefits of simulated negotiations generally to global health and global health law negotiations.”

- Thank you for reading the revised work. We are pleased the methodological changes addressed many of your concerns. We tried to maintain a balance between a description of this serious game and wider value of this pedagogical method of education. We agree with the reviewer that further research on serious games as capacity-building tools for global health diplomacy could be an important future avenue for advancing global health knowledge and practices.

“This article would benefit from discussion of literature on the problem-based learning benefits of simulation exercises in other international lawmaking contexts and reasoning by analogy – for example, there is ample literature on the use of Model UN simulation exercises. Connections to this literature would support the authors’ assessment of the pandemic agreement simulation.”

- We appreciate these suggestions and have further contextualized our analysis with regard to model UN exercises and problem-based learning in the introduction, including further references to the law literature (see lines 76-86 as well as other revisions in the introduction and discussion).
- We appreciate the connections drawn between our analysis and the use of simulations to explore and experience other areas of international law through different multilateral UN organizations. For the purposes of our analysis, both the course and pandemic treaty negotiation game were not intended or facilitated for learning or practices specific to global health law. Instead, the approach, pedagogy and methodologies adopted for this research and analysis drawn on broader, cross-disciplinary social sciences approaches and perspectives relevant to global health governance and policy.

Additional primary research could bolster the findings of this article, potentially including a survey of courses within the field of global health law that include simulation exercises (mindful of any ethical / privacy considerations there).

- Thank you for this suggestion for valuable future research. This is unfortunately beyond the scope of this article

The article would be strengthened by a broader discussion of why these simulation exercises are specifically useful for the teaching of global health as a field. The authors’ analysis on the alignment of these exercises with goals of decolonizing global health is useful, as is the analysis identifying global health challenges as wicked problems. More could be provided on the benefit of these exercises in light of the revitalization of global health lawmaking to undertake the reforms the article describes (the pandemic agreement and the IHR reforms).

- We have aimed to more clearly emphasize the utility of these exercises to global health in the discussion, including referencing current developments in global health such as the pandemic treaty negotiations. However, as noted above, as our focus was not specifically on global health law, we have been careful not to overstate contributions to this field. (see lines 772-785)
